# Association of Comprehensive Geriatric Assessment with Knowledge- and Technique-Related Eye Drop Adherence Problems in Glaucoma Assessed Using the Shimane University Glaucoma Eye Drop Adherence Questionnaire

**DOI:** 10.3390/jcm15135203

**Published:** 2026-07-03

**Authors:** Mayumi Furue, Yuya Kato, Hinako Ohtani, Chisako Ida, Kana Murakami, Mizuki Koike, Keigo Takagi, Yuto Yoshida, Kazunobu Sugihara, Masaki Tanito

**Affiliations:** Department of Ophthalmology, Shimane University Faculty of Medicine, Izumo 693-8501, Japan

**Keywords:** glaucoma, medication adherence, comprehensive geriatric assessment, cognitive impairment, eye drop instillation technique

## Abstract

**Background/Objectives**: Glaucoma medication adherence is influenced by multiple factors, including treatment-related knowledge, eye drop instillation technique, cognitive function, and systemic health status. However, the relationships between comprehensive geriatric assessment (CGA) results and specific adherence-related problems remain poorly understood. This study investigated the associations between CGA results and glaucoma eye drop adherence-related problems using the newly developed Shimane University Glaucoma Eye Drop Adherence Questionnaire (SU-GAQ). **Methods**: This retrospective study included 187 consecutive glaucoma patients who underwent CGA and completed the SU-GAQ at Shimane University Hospital. The SU-GAQ consists of Knowledge (Q1–Q5) and Technique (Q6–Q15) domains. CGA included the Mini-Cog, G8, and Age-Adjusted Charlson Comorbidity Index (ACCI). Associations between questionnaire responses and CGA results were evaluated using Spearman’s rank correlation and generalized regression analyses. Exploratory factor analysis (EFA) based on tetrachoric correlations was performed to evaluate the questionnaire structure. **Results**: Lower Mini-Cog scores were significantly associated with higher Knowledge-domain scores (ρ = −0.27, *p* = 0.0002) and higher overall questionnaire scores (ρ = −0.23, *p* = 0.002). In multivariate analysis, Mini-Cog remained independently associated with the Knowledge domain (estimate = −0.31, *p* < 0.0001) and total score (estimate = −0.44, *p* = 0.002). Higher ACCI scores were associated with higher Technique-domain scores (ρ = 0.19, *p* = 0.009) and remained significant in multivariate analysis (estimate = 0.16, *p* = 0.02). G8 scores were not significantly associated with questionnaire outcomes. EFA identified a two-factor structure corresponding to the predefined Knowledge and Technique domains, accounting for 71.2% of the total variance. **Conclusions**: Lower cognitive function was primarily associated with knowledge-related barriers to glaucoma medication adherence, whereas greater comorbidity burden was associated with technical difficulties in eye drop instillation. The SU-GAQ demonstrated a clinically meaningful two-domain structure and may facilitate identification of specific adherence-related barriers in glaucoma patients.

## 1. Introduction

Glaucoma is one of the leading causes of irreversible visual impairment worldwide and requires lifelong management to prevent progressive visual field loss [1,2]. A meta-analysis estimated that glaucoma affects 3.54% of individuals aged 40–80 years globally, corresponding to approximately 76 million people in 2020, with the number projected to increase to 111.8 million by 2040 [1]. Because glaucoma is typically asymptomatic until advanced stages, successful treatment depends largely on patients’ adherence to prescribed therapy. Topical intraocular pressure (IOP)-lowering medications remain the first-line treatment for many patients with glaucoma [3]; however, poor adherence to eye drop therapy is common and has been recognized as a major obstacle to achieving optimal treatment outcomes [4]. Adherence to glaucoma medications is a fundamental challenge in glaucoma care, with up to half of patients failing to achieve the intended benefits of treatment [4,5]. Long-term topical therapy may also be associated with ocular surface disease and other treatment-related adverse effects. Ocular surface disease has been reported in approximately 49–59% of patients receiving topical anti-glaucoma medications and is associated with reduced treatment satisfaction, poorer adherence, and diminished quality of life [6].

Medication adherence in glaucoma is a multifactorial behavior. Common barriers to adherence include forgetfulness, difficulty with eye drop administration, treatment complexity, medication side effects, and inadequate understanding of glaucoma and its treatment [7,8]. These barriers may broadly reflect deficits in either treatment-related knowledge or practical eye drop instillation skills, both of which can compromise treatment effectiveness [7,8]. Importantly, adherence in glaucoma differs from adherence to oral medications because patients must not only remember to use the medication but also possess the practical skills required for successful eye drop instillation. Poor eye drop technique is a significant impediment to achieving adequate intraocular pressure control and may reduce treatment effectiveness even among patients who are otherwise adherent to therapy [9,10,11]. Older patients frequently experience practical difficulties with self-instillation of glaucoma eye drops because of age-related physical limitations and systemic comorbidities [12]. In our previous study, older age, decreased cognitive function, hyperopia, and reduced foveal sensitivity were identified as risk factors for failed eye drop instillation [13]. These observations suggest that glaucoma adherence encompasses at least two distinct but related components: understanding of treatment and the ability to correctly administer eye drops.

Cognitive impairment has been recognized as an important barrier to medication adherence in elderly patients with chronic diseases [14,15]. In glaucoma, lower cognitive function has been associated with more severe structural and functional glaucomatous damage independent of age and visual acuity [16]. Furthermore, glaucoma patients with lower cognitive function scores exhibit more advanced visual field loss and retinal structural abnormalities [17], and cognitive impairment may reduce the reliability of visual field testing [18]. Collectively, these findings suggest that cognitive decline may influence multiple aspects of glaucoma management. However, it remains unclear whether cognitive dysfunction primarily affects patients’ understanding of glaucoma treatment, their practical ability to administer eye drops, or both.

The aging of the glaucoma population further emphasizes the importance of understanding age-related factors that may influence glaucoma medication management. Cognitive decline, frailty, and systemic comorbidities become increasingly prevalent with advancing age and may affect patients’ ability to perform complex self-care behaviors. Comprehensive geriatric assessment (CGA) is a multidimensional, usually interdisciplinary, diagnostic process intended to evaluate an older person’s medical, psychosocial, and functional capabilities and to develop a coordinated plan for treatment and long-term follow-up [19]. Among commonly used CGA tools, the Mini-Cog is a brief screening test for cognitive impairment [20], the G8 is a screening tool for frailty and geriatric vulnerability [21], and the Charlson Comorbidity Index (CCI) quantifies systemic disease burden and age-related health risk [22,23]. Although these assessments are widely used in geriatric medicine, their relationship with specific problems related to glaucoma eye drop adherence has not been fully investigated.

Several glaucoma-specific adherence assessment tools have previously been developed, including the Glaucoma Treatment Compliance Assessment Tool (GTCAT) and the Glaucoma Medication Adherence Self-Efficacy Questionnaire (GMASQ) [24,25]. These instruments provide valuable information regarding adherence-related behaviors, beliefs, and self-efficacy; however, they do not specifically distinguish treatment-related knowledge deficits from practical eye drop instillation difficulties. As these barriers may require different clinical interventions, a tool capable of separately identifying these domains may provide more clinically actionable information in routine glaucoma care. To better characterize adherence-related difficulties encountered in routine clinical practice, we developed the Shimane University Glaucoma Eye Drop Adherence Questionnaire (SU-GAQ). The questionnaire was developed based on evidence that successful glaucoma medication adherence depends not only on patients’ understanding of glaucoma treatment but also on their ability to correctly instill eye drops [7,8,9,10,11]. Accordingly, the SU-GAQ consists of two conceptual domains: a Knowledge domain evaluating understanding of glaucoma treatment and a Technique domain assessing practical difficulties related to eye drop instillation. By separately assessing these domains, the questionnaire may provide more clinically actionable information than conventional adherence assessments and facilitate the identification of specific barriers that can be addressed through targeted interventions. The purpose of the present study was to investigate the associations between CGA results and responses to the SU-GAQ in patients with glaucoma. In addition, we sought to evaluate the underlying structure of the questionnaire using exploratory factor analysis. We hypothesized that different components of CGA would be associated with different types of adherence-related problems and that the questionnaire would demonstrate a two-domain structure corresponding to Knowledge and Technique.

## 2. Materials and Methods

### 2.1. Study Design and Subjects

This retrospective study adhered to the principles of the Declaration of Helsinki, and the Institutional Review Board (IRB) of Shimane University Hospital reviewed and approved the research (IRB No. 20200228-2, issued on 26 March 2020, with an updated protocol issued on 27 April 2026). IRB approval did not require written informed consent from each patient for publication. Instead, the study protocol was posted at the study institutions to provide participants with the opportunity to opt out of the study. This study included 187 consecutive Japanese subjects (103 males and 84 females) who visited the glaucoma outpatient clinic of Shimane University Hospital between June 2023 and June 2025, underwent an assessment of glaucoma eye drop adherence using the SU-GAQ, and received a CGA, including the Mini-Cog, G8, and CCI. Patients with incomplete SU-GAQ or CGA (Mini-Cog, G8, or CCI) data were to be excluded from the analysis; however, no such cases were identified during the study period. In our glaucoma outpatient clinic, assessment of glaucoma eye drop adherence and CGA are routinely performed as part of the initial evaluation at the first visit or during preoperative examinations.

### 2.2. Data Collection

We retrospectively reviewed the medical records and collected data on age at the time of CGA, sex, presence of hypertension (HT), presence of diabetes mellitus (DM), history of cataract surgery, glaucoma subtype, results of the SU-GAQ, and scores of the three CGA tests. The presence of HT and DM was determined based on self-reported medical history and the use of systemic medications. In all patients, one author (M.T.) performed a comprehensive ophthalmic examination, including IOP measurement, slit-lamp examination, fundus examination, gonioscopic examination, and visual field testing, and determined the glaucoma subtype according to the definitions provided in the Japan Glaucoma Society Guidelines for Glaucoma, 5th edition [26]. Glaucoma subtypes were classified as primary open-angle glaucoma (POAG), primary angle-closure disease (PACD), exfoliation glaucoma (EXG), and other types of glaucoma (others). Normal tension glaucoma was classified as POAG. Exfoliation syndrome was classified as EXG. Primary angle-closure glaucoma, primary angle-closure, and primary angle-closure suspect were classified as PACD. Subject-based classification was performed hierarchically. Subjects were classified as “others” if at least one eye was diagnosed with a glaucoma subtype categorized as others. Among the remaining subjects, those with at least one eye diagnosed with EXG were classified as EXG. Among the remaining subjects, those with at least one eye diagnosed with PACD were classified as PACD. The remaining subjects were classified as POAG. Therefore, the POAG group included subjects with bilateral POAG as well as subjects with unilateral POAG and a normal fellow eye. Subjects were classified as having a history of cataract surgery if cataract surgery had been performed in at least one eye.

### 2.3. SU-GAQ

The questionnaire consisted of 15 items (Table 1). Questions 1–5 comprised five questions regarding knowledge of glaucoma and glaucoma pharmacotherapy (Knowledge domain), whereas Questions 6–15 comprised 10 questions regarding eye drop instillation technique (Technique domain). All items were structured in a checklist format in which participants checked the items that applied to them. The questionnaire was administered using a combination of self-completion and interview-based confirmation. First, the questionnaire was provided to the patients and completed by the patients themselves. Subsequently, ophthalmic nurses used the questionnaire as a reference during eye drop instruction sessions and performed a double-check to confirm the patients’ understanding of the questions and to identify any missing responses. For statistical analyses, responses were treated as binary yes/no variables. All questionnaires and interviews were conducted in Japanese. The original Japanese version of the SU-GAQ is provided in Table S1 of the Supplementary Materials, and the original printed format of the questionnaire is provided as Figure S1.

### 2.4. CGA Tests

The G8 and CCI evaluations were carried out by ophthalmologists, whereas ophthalmic nurses administered the Mini-Cog test during educational sessions on glaucoma eye drop instillation. All evaluations were performed in person using an interview-based format.

Mini-Cog: Cognitive status was evaluated using the Mini-Cog test [20]. This screening tool combines a three-word delayed recall task with a clock-drawing task to assess memory and executive function. Scores range from 0 to 5, with scores of 0–2 indicating possible cognitive impairment and scores of 3–5 considered within the normal range. The assessment can generally be completed within approximately three minutes.

G8 Screening Tool: Frailty and geriatric vulnerability were assessed using the G8 screening tool [21]. The instrument contains eight components evaluating nutritional decline during the preceding three months, recent involuntary weight loss, reduced mobility, neuropsychological problems, low body mass index (BMI ≤ 21), use of multiple medications (three or more drugs per day), subjective health perception, and older age (≥85 years). The overall score ranges from 0 to 17, and a score of 14 or lower is generally considered suggestive of frailty requiring further geriatric assessment.

Age-Adjusted Charlson Comorbidity Index (ACCI): Comorbidity burden was quantified using the CCI [22]. The index assigns weighted values to a range of chronic systemic diseases, including cardiovascular, cerebrovascular, pulmonary, hepatic, renal, malignant, and metabolic disorders, as well as dementia and AIDS. Higher total scores indicate greater systemic disease burden and a higher estimated risk of long-term mortality. To account for aging, additional age-related points were incorporated into the original CCI score according to the ACCI method: 1 point for ages 41–50 years, 2 points for 51–60 years, 3 points for 61–70 years, 4 points for 71–80 years, and 5 points for ≥81 years [23]. This age adjustment improves risk stratification in elderly populations by integrating both comorbidity load and age-associated vulnerability.

### 2.5. Statistical Analysis

All continuous data were expressed as mean ± standard deviation (SD) with 95% confidence intervals (CIs), while categorical data were presented as counts and percentages. Associations between SU-GAQ variables (Q1–Q15, Knowledge, Technique, and Q_Total) and CGA test scores or other background parameters were evaluated using Spearman’s rank correlation coefficient (ρ). Spearman correlation was selected because questionnaire items were binary variables and summary scores were ordinal or discrete count variables. Correlation coefficients (ρ) and corresponding *p*-values were calculated for each pairwise comparison. To account for multiple comparisons across all tested associations, *p*-values were adjusted using the Benjamini–Hochberg false discovery rate (FDR) correction. An adjusted *p*-value < 0.05 was considered statistically significant. Results were visualized using a heatmap in which color intensity represented the magnitude and direction of Spearman’s correlation coefficient. The associations between SU-GAQ responses and various background parameters were also assessed using generalized regression models. Because the Knowledge, Technique, and Total scores represented summed counts across multiple questionnaire items and showed sufficient variability within the study population, they were analyzed as continuous outcome variables in the generalized regression models. In this model, the three CGA test results and sex were included as explanatory variables. Age was not included because age is incorporated into the G8 and ACCI calculation and is also strongly related to CGA measures. Inclusion of age together with these variables could introduce collinearity and complicate interpretation of the independent effects of CGA parameters. Internal consistency was evaluated separately for the Knowledge and Technique domains using Cronbach’s alpha coefficient, with higher values indicating greater internal reliability among items within each domain.

To examine the latent structure of the questionnaire, an exploratory factor analysis (EFA) was conducted using tetrachoric correlations, which are appropriate for binary response data and assume underlying continuous latent variables [27]. Eigenvalues were derived from the tetrachoric correlation matrix, and the number of factors was determined based on the Kaiser criterion (eigenvalue > 1) and inspection of the scree plot [28]. Factor loadings were extracted and orthogonally rotated using the varimax method to facilitate interpretability by achieving a simpler and more interpretable factor structure. Based on the predefined conceptual classification of items into Knowledge (Q1–Q5) and Technique (Q6–Q15) domains, the resulting factor structure was interpreted in light of this framework. All statistical analyses were performed using JMP Student Edition version 19.1.1 (SAS Institute, Inc., Cary, NC, USA) and Python-based statistical tools, including the SciPy and statsmodels libraries.

## 3. Results

The demographic characteristics of the patients, including age, sex, presence of hypertension and diabetes mellitus, history of cataract surgery, glaucoma subtype, scores of the three CGA tests (Mini-Cog, G8, and ACCI), and medication score, are presented in Table 2. The mean age of the participants was 70.0 ± 13.1. HT and DM were present in 103 subjects (55%) and 30 subjects (16%). A history of cataract surgery was recorded in 81 subjects (43%). Regarding the glaucoma subtype, 109 subjects (58%) had POAG, 9 (5%) had PACD, 39 (21%) had EXG, and 30 (16%) had other types of glaucoma. Regarding the three CGA scores, the mean total Mini-Cog score was 4.3 ± 1.0, the mean G8 screening tool score was 14.2 ± 2.1, and the mean ACCI was 3.8 ± 1.5.

The responses to the SU-GAQ are presented in Table 3. In total, at least one ‘Yes’ response was identified in 89 subjects (48%). In the Knowledge domain (i.e., Q1-Q5), at least one “Yes” response was identified in 52 subjects (28%). The most frequent issue was Q2, reported by 40 subjects (21%), followed by Q3, reported by 22 subjects (12%). In the Technique domain (i.e., Q6-Q15), at least one ‘Yes’ response was identified in 62 subjects (33%). The most frequent technical difficulty was Q12, reported by 34 subjects (18%), followed by Q9, reported by 31 subjects (17%). Internal consistency analysis demonstrated acceptable reliability for both questionnaire domains. Cronbach’s alpha was 0.757 for the Knowledge domain (Q1–Q5) and 0.744 for the Technique domain (Q6–Q15), supporting the internal consistency of the predefined domain structure.

Table 4 presents the associations between responses to each SU-GAQ item and CGA parameters. Lower Mini-Cog scores were significantly correlated with higher overall total scores (ρ = −0.23, *p* = 0.002) and higher knowledge domain total scores (ρ = −0.27, *p* = 0.0002). At the individual item level, lower Mini-Cog scores were correlated with higher scores in Q1 (ρ = −0.25, *p* = 0.0006), Q2 (ρ = −0.31, *p* = 0.00002), Q4 (ρ = −0.17, *p* = 0.02), Q7 (ρ = −0.22, *p* = 0.002), Q12 (ρ = −0.14, *p* = 0.05), and Q15 (ρ = −0.17, *p* = 0.02). G8 scores were not significantly correlated with the overall total (ρ = −0.09, *p* = 0.20), knowledge domain total (ρ = 0.04, *p* = 0.63), technique domain total (ρ = −0.12, *p* = 0.11), or any of the individual items (Q1–Q15). Higher ACCI scores were significantly correlated with higher overall total scores (ρ = 0.19, *p* = 0.009) and higher technique domain total scores (ρ = 0.19, *p* = 0.009). At the individual level, higher ACCI scores were correlated with higher scores in Q8 (ρ = 0.20, *p* = 0.006), Q9 (ρ = 0.15, *p* = 0.04), Q10 (ρ = 0.17, *p* = 0.02), and Q12 (ρ = 0.16, *p* = 0.03).

Table 5 shows the associations between the SU-GAQ responses and various background parameters, including Age, Sex, HT, DM, Cataract surgery history, and Glaucoma type. Older age was significantly correlated with higher overall total scores (ρ = 0.23, *p* = 0.002) and higher technique domain total scores (ρ = 0.23, *p* = 0.001). At the individual item level, older age was significantly correlated with higher scores in Q8 (ρ = 0.20, *p* = 0.007), Q9 (ρ = 0.18, *p* = 0.02), Q11 (ρ = 0.19, *p* = 0.009), Q12 (ρ = 0.17, *p* = 0.02), and Q15 (ρ = 0.16, *p* = 0.03). No significant correlations were observed between sex and any of the SU-GAQ summary metrics or individual items. The presence of HT was only significantly correlated with a higher score in Q12 (ρ = 0.15, *p* = 0.04). The presence of DM was significantly correlated with higher overall total scores (ρ = 0.17, *p* = 0.02) and higher technique domain total scores (ρ = 0.21, *p* = 0.004). At the individual item level, DM was correlated with higher scores in Q9 (ρ = 0.16, *p* = 0.03), Q10 (ρ = 0.23, *p* = 0.001), and Q12 (ρ = 0.25, *p* = 0.001). Having a history of cataract surgery was significantly correlated with higher technique domain total scores (ρ = 0.18, *p* = 0.02), as well as higher scores in Q9 (ρ = 0.16, *p* = 0.03), Q10 (ρ =0.23, *p* = 0.001), and Q11 (ρ = 0.21, *p* = 0.005). Regarding glaucoma type, the POAG phenotype was significantly correlated with higher technique domain total scores (ρ = 0.16, *p* = 0.03). At the individual item level, the non-POAG phenotype was significantly correlated with higher scores in Q9 (ρ = 0.26, *p* = 0.0003), Q10 (ρ = 0.25, *p* = 0.001), and Q11 (ρ = 0.22, *p* = 0.003). Knowledge domain total was not significantly correlated with any of the background parameters examined.

**Figure 1:**
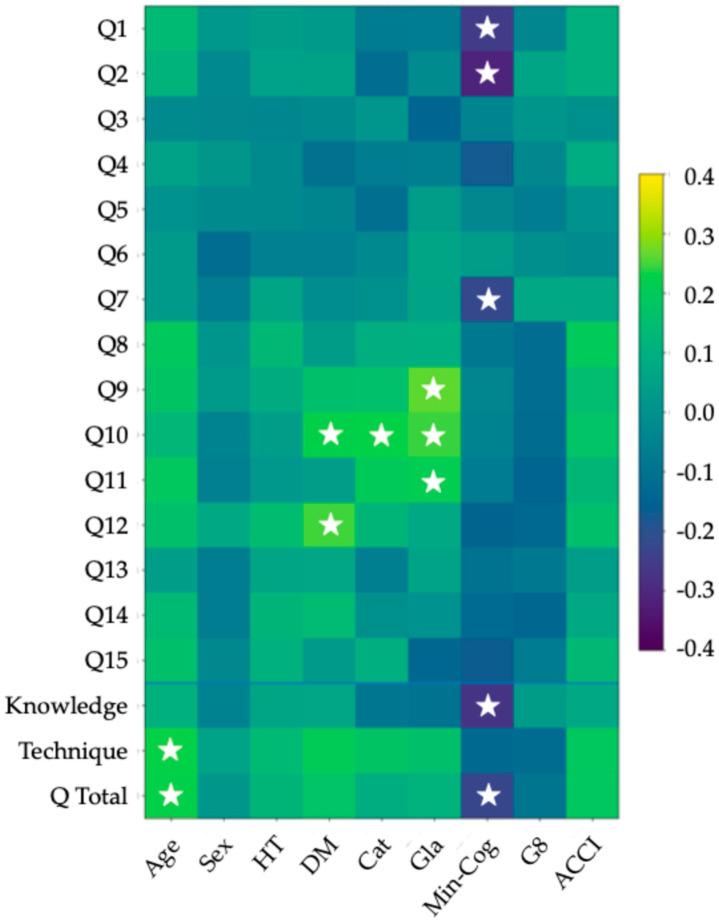
Spearman correlation heatmap of questionnaire items and clinical variables with FDR correction. Heatmap showing Spearman correlation coefficients (ρ) between questionnaire items (Q1–Q15), summary scores (Knowledge, Technique, and Q Total), and clinical variables (age, sex, hypertension [HT], diabetes mellitus [DM], cataract [Cat], glaucoma subtype [Gla], Mini-Cog, G8, and ACCI). Sex was coded as 0 = male and 1 = female. HT, DM, and Cat were coded as 0 = no and 1 = yes. Glaucoma subtype was coded as 0 = POAG and 1 = non-POAG (EXG, AC, or other). Color intensity represents the magnitude and direction of Spearman’s correlation coefficient (ρ), with warmer colors indicating positive correlations and cooler colors indicating negative correlations. Stars (★) indicate statistically significant associations after false discovery rate (FDR) correction using the Benjamini–Hochberg method (adjusted *p* < 0.05). A horizontal line separates individual questionnaire items (Q1–Q15) from summary scores (Knowledge, Technique, and Q Total).

Figure 1 shows the results of the multiple-comparison adjustment using the Benjamini–Hochberg method for the univariate comparisons presented in Table 4 and Table 5. Color intensity represents the magnitude and direction of Spearman’s correlation coefficient, with warmer colors indicating positive correlations and cooler colors indicating negative correlations. Stars (★) indicate statistically significant associations after adjustment (adjusted *p* < 0.05). After adjustment, significant associations were observed between age and the technique domain total score as well as the overall total score; between DM and Q10 and Q12; between cataract surgery and Q10; between glaucoma subtype and Q9–Q11; and between Mini-Cog score and the knowledge domain total score, overall total score, Q1, Q2, and Q7. No statistically significant associations were observed for the other comparison pairs after adjustment.

Table 6 shows the results of multivariate analysis of the associations between SU-GAQ responses and CGA scores. In the model, lower Mini-Cog scores were significantly associated with higher scores in the knowledge domain (estimate = −0.31, 95% CI: −0.47 to −0.16, *p* < 0.0001) and a higher total score (estimate = −0.44, 95% CI: −0.72 to −0.16, *p* = 0.002). Higher ACCI scores were significantly associated with higher scores in the technique domain (estimate = 0.16, 95% CI: 0.02 to 0.31, *p* = 0.02). The remaining parameters showed no significant associations with any of the SU-GAQ outcomes.

**Figure 2:**
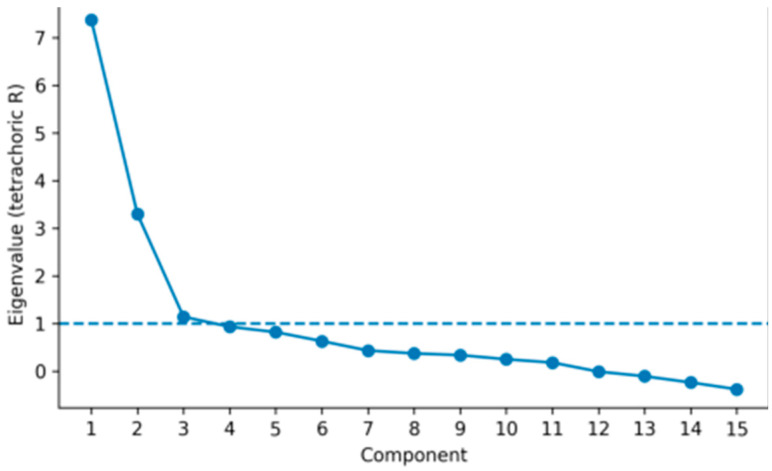
Scree plot based on tetrachoric correlations. Scree plot of eigenvalues derived from the tetrachoric correlation matrix of Q1–Q15. The plot shows a clear inflection after the second factor, supporting a two-factor structure corresponding to Knowledge and Technique domains.

**Figure 3:**
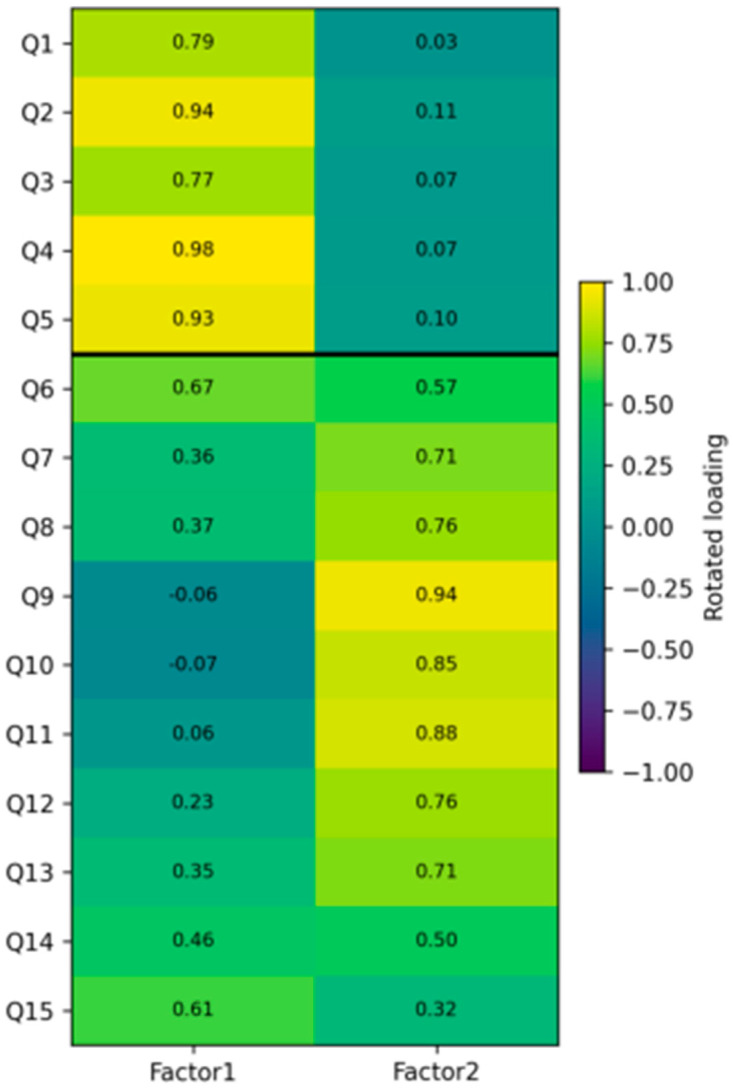
Heatmap of varimax-rotated factor loadings from the two-factor exploratory factor analysis using tetrachoric correlations. Knowledge items (Q1–Q5) load predominantly on Factor 1, whereas Technique items (Q6–Q15) load primarily on Factor 2. A horizontal line separates the two domains.

Exploratory factor analysis based on tetrachoric correlations was performed to evaluate whether the questionnaire items formed meaningful underlying domains. Eigenvalues and explained variance derived from the tetrachoric correlation matrix are summarized in Table 7. The first and second factors had eigenvalues of 7.38 and 3.30, respectively, together accounting for 71.2% of the total variance, indicating that these two factors captured the majority of the questionnaire structure. The scree plot (Figure 2) showed a clear inflection point after the second factor, supporting a two-factor solution. Varimax-rotated factor loadings are presented in Table 8. All Knowledge items (Q1–Q5) loaded strongly on Factor 1 with minimal loadings on Factor 2, whereas most Technique items (Q7–Q13) showed high loadings on Factor 2. Several items, including Q6, Q14, and Q15, exhibited moderate loadings on both factors, indicating partial overlap between the domains. The heatmap of factor loadings (Figure 3) visually demonstrated the general separation between Knowledge-related and Technique-related items while also illustrating cross-loading patterns. Overall, the observed factor structure was consistent with the predefined classification of the questionnaire into Knowledge and Technique domains and supported the interpretation that these domains represent distinguishable, though not entirely independent, latent constructs.

## 4. Discussion

The present study evaluated the associations between CGA results and glaucoma eye drop adherence-related problems using a newly developed glaucoma-specific questionnaire, the SU-GAQ. Three major findings emerged. First, lower Mini-Cog scores were associated with a higher frequency of knowledge-related problems regarding glaucoma treatment and eye drop use. Second, higher ACCI scores were associated with a greater number of technique-related difficulties in eye drop instillation. Third, exploratory factor analysis supported the predefined two-domain structure of the questionnaire, consisting of Knowledge and Technique domains. These findings suggest that cognitive impairment and systemic comorbidity burden may influence different aspects of glaucoma medication management and provide preliminary support for evaluating these domains separately using the SU-GAQ. This interpretation is consistent with recent evidence indicating that glaucoma medication adherence is influenced by multiple patient-related factors and clinical characteristics [29].

The association between lower Mini-Cog scores and higher Knowledge-domain scores was one of the most consistent findings in this study. Patients with lower cognitive function were more likely to report uncertainty regarding the purpose of glaucoma medications, the identity of pressure-lowering eye drops, and the prescribed dosing schedule. Previous studies have suggested that cognitive impairment adversely affects self-management abilities and treatment adherence in chronic diseases, including glaucoma [14,15,30]. In addition, lower cognitive function has been associated with more severe glaucomatous damage and reduced reliability of visual field testing [16,17,18]. The present findings extend these observations by suggesting that cognitive decline primarily affects treatment-related knowledge rather than eye drop instillation technique. Because successful glaucoma management depends on patients’ understanding of the chronic nature of the disease and the importance of continuous therapy [4,7], patients with reduced Mini-Cog scores may benefit from simplified educational materials, repeated instruction, involvement of family members or caregivers, and more frequent reinforcement of treatment information [8].

In contrast, higher ACCI scores were associated mainly with Technique-domain problems. Patients with greater systemic comorbidity burden were more likely to report difficulties related to accurate eye drop placement, visualization of the bottle tip, and bottle-tip contact with the eyelids or eyelashes. These findings are clinically plausible because patients with multiple systemic diseases frequently experience reduced physical function, impaired manual dexterity, tremor, visual impairment, and other limitations that may interfere with successful eye drop administration [10,11,12]. Similar associations were also observed for older age, diabetes mellitus, and previous cataract surgery, which were primarily linked to Technique-domain items. Our previous study likewise identified older age as a risk factor for failed eye drop instillation [13]. Together, these findings suggest that practical difficulties in eye drop administration may be influenced more strongly by physical and systemic factors than by cognitive factors. From a clinical perspective, identifying such patients may facilitate targeted interventions, including instillation aids, caregiver support, alternative medication delivery strategies, or surgical treatment when appropriate [12].

Interestingly, the G8 screening tool was not significantly associated with either the Knowledge or Technique domains. The G8 is a well-established screening instrument for frailty and geriatric vulnerability and has been validated in older populations [21,31]. However, because its components primarily assess nutritional status, mobility, medication burden, self-perceived health, and age, it may be less sensitive to the specific cognitive and practical skills required for successful glaucoma medication management. This may explain the absence of significant associations observed in the present study. No significant associations were observed between sex and any of the SU-GAQ outcomes. However, the present study was not specifically designed or powered to detect sex-related differences, and further investigation may be warranted.

Exploratory factor analysis based on tetrachoric correlations demonstrated a clear two-factor structure that broadly corresponded to the predefined Knowledge and Technique domains, which is consistent with the use of exploratory factor analysis to identify underlying latent constructs in questionnaire-based research [32]. Knowledge items (Q1–Q5) loaded predominantly on Factor 1, whereas most Technique items (Q7–Q13) loaded on Factor 2. At the same time, several items (Q6, Q14, and Q15) exhibited cross-loadings on both factors, suggesting that not all questionnaire items fall neatly into a single domain. Clinically, this finding is intuitive because successful glaucoma eye drop administration often requires both adequate treatment-related knowledge and appropriate practical skills. For example, forgetting eye drop instillation (Q15) may reflect not only memory or behavioral issues but also insufficient understanding of the importance of continuous treatment. Similarly, post-instillation care such as wiping excess medication (Q14) may depend on both procedural habits and knowledge of proper instillation techniques. Therefore, the observed overlap does not necessarily indicate a weakness of the questionnaire but rather reflects the interconnected nature of knowledge and technique in real-world glaucoma self-management. Nevertheless, the overall pattern was highly consistent with the conceptual framework used during questionnaire development [33]. The two-factor solution explained more than 70% of the total variance, indicating that the majority of response variability could be understood in terms of these two latent domains. These findings provide preliminary support for the construct validity of the SU-GAQ and suggest that glaucoma eye drop adherence problems may be meaningfully categorized into deficiencies in treatment-related knowledge and difficulties in instillation technique. From a practical standpoint, this distinction may be useful because the interventions required to address these problems differ substantially. The use of tetrachoric correlations further strengthens this interpretation because this approach is specifically designed for binary questionnaire data and assumes that observed yes/no responses arise from underlying continuous latent traits [27,28]. Therefore, the identified factor structure is likely to reflect meaningful latent dimensions rather than merely patterns of observed categorical responses. The internal consistency of both domains was acceptable, with Cronbach’s alpha values of 0.757 for the Knowledge domain and 0.744 for the Technique domain. These findings support the construct validity of the SU-GAQ and suggest that adherence-related problems in glaucoma may be meaningfully categorized into deficiencies in treatment-related knowledge and difficulties in instillation technique. An important consideration is that the SU-GAQ evaluates self-reported knowledge deficits and eye drop instillation difficulties rather than objectively measured medication adherence. Therefore, the present findings should be interpreted as reflecting adherence-related problems, and further studies incorporating objective adherence measures, such as electronic monitoring systems or pharmacy refill records, are needed to determine how these domains relate to actual medication use.

This study has several limitations that should be considered when interpreting the findings. First, this was a retrospective single-center study involving Japanese patients treated at a university-based glaucoma clinic, which may limit the generalizability of the findings to other populations and healthcare settings. In addition, no formal a priori sample size calculation was performed because all consecutive eligible patients during the study period were included. Therefore, the study may have been underpowered to detect some associations, and non-significant findings should be interpreted with caution because of the potential for type II error. Second, the questionnaire responses were self-reported and may therefore be influenced by recall bias or social desirability bias. Third, although tetrachoric correlations and exploratory factor analysis are appropriate for binary questionnaire data, the identified factor structure remains exploratory and should be confirmed in independent populations using confirmatory factor analysis. Fourth, several items demonstrated moderate cross-loadings, suggesting that knowledge and technique are not entirely independent constructs and that further refinement of some questionnaire items may improve construct clarity. Finally, the present study did not evaluate objective adherence measures, such as electronic monitoring systems, pharmacy refill records, or direct observation of eye drop administration. Consequently, the relationship between questionnaire scores and actual medication adherence remains to be determined. In addition, test–retest reliability, convergent validity with established adherence instruments, and discriminant validity were not evaluated in the present study and should be investigated in future validation studies. Although medication adherence is increasingly recognized as a multifaceted construct that includes treatment-related knowledge and eye drop instillation skills, the present questionnaire primarily evaluates self-reported adherence-related problems rather than objectively measured adherence behavior. Accordingly, the present findings should be interpreted as providing preliminary psychometric evidence rather than a comprehensive validation of the SU-GAQ.

From a practical perspective, routine administration of CGA instruments such as the Mini-Cog, G8, and ACCI may not be feasible for all patients in busy ophthalmology clinics. The present findings suggest that responses to the SU-GAQ may reflect underlying cognitive and comorbidity-related factors identified by CGA. Therefore, the SU-GAQ may serve as a simple screening tool to identify patients who are likely to benefit from more detailed geriatric assessment or targeted adherence-related interventions. Patients with predominantly Knowledge-domain problems may benefit from enhanced educational support, whereas those with predominantly Technique-domain problems may require instillation training, assistive devices, caregiver involvement, or consideration of alternative treatment strategies.

## 5. Conclusions

Lower Mini-Cog scores were associated primarily with knowledge-related problems regarding glaucoma treatment, whereas higher ACCI scores were associated mainly with technical difficulties in eye drop instillation. In contrast, G8 scores showed no significant association with questionnaire outcomes. Exploratory factor analysis provided preliminary support for the proposed two-domain structure consisting of Knowledge and Technique domains. These findings suggest that different components of geriatric assessment may influence distinct aspects of glaucoma medication management and support the use of targeted interventions according to the underlying cause of adherence-related problems. In clinical practice, assessment of cognitive function and comorbidity burden may help identify patients who would benefit from tailored educational support or assistance with eye drop administration. Future studies should validate the SU-GAQ in independent populations and investigate whether domain-specific interventions can improve long-term glaucoma medication adherence and treatment outcomes.

## Figures and Tables

**Table 1:** The Shimane University Glaucoma Eye Drop Adherence Questionnaire (SU-GAQ).

Item No.	Content
**Knowledge domain items**	
Q1	I do not know that glaucoma eye drops are medications that lower intraocular pressure.
Q2	When using multiple eye drops, I do not know which one lowers intraocular pressure.
Q3	When using multiple eye drops, I do not wait at least 5 min between instillations.
Q4	I do not know the prescribed frequency of my eye drops, or I sometimes use them the wrong number of times.
Q5	I am unsure about when to use my eye drops.
**Technique domain items**	
Q6	I am unable to tilt my head back when instilling eye drops.
Q7	I am unable to keep my eye fully open when instilling eye drops.
Q8	My hands tremble, making it difficult to instill the eye drops properly.
Q9	I miss the correct position when instilling the eye drops.
Q10	I cannot clearly see the tip of the bottle, making instillation difficult.
Q11	The eye drop often does not enter my eye with a single drop, so I instill multiple drops.
Q12	The tip of the bottle sometimes touches my eyelashes or eyelid during instillation.
Q13	After instillation, I do not close my eyes or press the inner corner of my eye (nasolacrimal occlusion).
Q14	After instillation, I do not wipe away or clean off excess medication around my eye.
Q15	I sometimes forget to instill my eye drops.

**Table 2:** Demographic Characteristics of the Subjects.

Parameter	N or Mean ± SD	95% CI or %
Subjects	187	
Age, years	70.0 ± 13.1	68.2, 71.9
Sex		
Male	103	55
Female	84	45
HT		
Yes	103	55
No	84	45
DM		
Yes	30	16
No	157	84
Cataract surgery		
Yes	81	43
No	106	57
Glaucoma type		
POAG	109	58
PACD	9	5
EXG	39	21
Others	30	16
Mini-Cog		
Word recall	2.4 ± 0.8	2.3, 2.5
Clock drawing	1.9 ± 0.4	1.9, 2.0
Mini-Cog total	4.3 ± 1.0	4.1, 4.4
G8		
G8_A	1.9 ± 0.3	1.9, 2.0
G8_B	2.8 ± 0.6	2.7, 2.9
G8_C	2.0 ± 0.2	1.9, 2.0
G8_E	1.9 ± 0.3	1.9, 2.0
G8_F	2.1 ± 1.0	2.0, 2.3
G8_H	0.6 ± 0.5	0.6, 0.7
G8_P	1.2 ± 0.8	1.1, 1.3
G8 Age	1.7 ± 0.7	1.6, 1.8
G8_total	14.2 ± 2.1	13.9, 14.5
CCI		
CCI_1	0.0 ± 0.1	−0.0, 0.0
CCI_2	0.0 ± 0.2	−0.0, 0.1
CCI_3	0 ± 0	0,0
CCI_4	0.0 ± 0.1	−0.0, 0.0
CCI_5	0.0 ± 0.1	−0.0, 0.0
CCI_6	0.0 ± 0.1	−0.0, 0.0
CCI_7	0.0 ± 0.2	0.0, 0.1
CCI_8	0 ± 0	0, 0
CCI_9	0.0 ± 0.3	0.0, 0.1
CCI_10	0 ± 0	0, 0
CCI_11	0.0 ± 0.2	−0.0, 0.1
CCI_12	0.0 ± 0.1	−0.0, 0.0
CCI_13	0.0 ± 0.1	−0.0, 0.0
CCI_14	0.1 ± 0.5	0.1, 0.2
CCI_15	0 ± 0	0, 0
CCI_16	0 ± 0	0, 0
CCI_17	0 ± 0	0, 0
CCI_total	0.3 ± 0.8	0.2, 0.4
ACCI total	3.8 ± 1.5	3.6, 4.0

SD, standard deviation; CI, confidence interval; HT, hypertension; DM, diabetes mellitus; POAG, primary open angle glaucoma; PACD, primary angle closure disease; EXG, exfoliation glaucoma; CCI, Charlson Comorbidity Index; ACCI, age-adjusted CCI.

**Table 3:** Responses to the SU-GAQ.

Item No.	Yes, n (%)	No, n (%)
Q1	21 (11)	166 (89)
Q2	40 (21)	147 (79)
Q3	22 (12)	165 (88)
Q4	10 (5)	177 (95)
Q5	10 (5)	177 (95)
Q6	3 (2)	184 (98)
Q7	7 (4)	180 (96)
Q8	4 (2)	183 (98)
Q9	31 (17)	156 (83)
Q10	16 (9)	171 (91)
Q11	19 (10)	168 (90)
Q12	34 (18)	153 (82)
Q13	7 (4)	180 (96)
Q14	7 (4)	180 (96)
Q15	15 (8)	172 (92)
Knowledge domain total	52 (28)	135 (72)
Technique domain total	62 (33)	125 (67)
Total	89 (48)	98 (52)

Knowledge domain, Q1–Q5; Technique domain, Q6–Q15.

**Table 4:** Univariate Analysis of the Associations Between SU-GAQ Responses and CGA Test Results.

Parameter	Mini-Cog		G8		ACCI	
	ρ	*p*-Value	ρ	*p*-Value	ρ	*p*-Value
Q1	−0.25	0.0006 **	−0.04	0.62	0.10	0.17
Q2	−0.31	0.00002 **	0.06	0.39	0.10	0.16
Q3	−0.04	0.56	0.01	0.85	0.00	0.98
Q4	−0.17	0.02	−0.03	0.68	0.09	0.22
Q5	−0.03	0.68	−0.06	0.38	0.01	0.92
Q6	0.04	0.59	0.00	0.96	−0.02	0.78
Q7	−0.22	0.002 **	0.07	0.32	0.07	0.35
Q8	−0.08	0.28	−0.11	0.12	0.20	0.006 **
Q9	−0.04	0.61	−0.11	0.12	0.15	0.04 *
Q10	−0.04	0.55	−0.12	0.11	0.17	0.02 *
Q11	−0.07	0.34	−0.14	0.06	0.12	0.09
Q12	−0.14	0.05	−0.13	0.08	0.16	0.03 *
Q13	−0.10	0.17	−0.08	0.28	0.04	0.57
Q14	−0.12	0.10	−0.13	0.07	0.08	0.27
Q15	−0.17	0.02 *	−0.07	0.31	0.13	0.07
Knowledge domain total	−0.27	0.0002 **	0.04	0.63	0.08	0.30
Technique domain total	−0.12	0.09	−0.12	0.11	0.19	0.009 **
Total	−0.23	0.002 **	−0.09	0.20	0.19	0.009 **

*p*-values are calculated using Spearman’s rank correlation coefficient. * and ** indicate *p* < 0.05 and *p* < 0.01, respectively. ρ, Spearman’s rank correlation coefficient. ACCI, age-adjusted Charlson Comorbidity Index.

**Table 5:** Univariate Analysis of the Associations Between SU-GAQ Responses and various background parameters.

Item No.	Age (/Year)		Sex (F/M)		HT (Y/N)		DM (Y/N)		Cataract Surgery (Y/N)		Glaucoma Type (Non-POAG/POAG)	
	ρ	*p*-Value	ρ	*p*-Value	ρ	*p*-Value	ρ	*p*-Value	ρ	*p*-Value	ρ	*p*-Value
Q1	0.14	0.052	0.02	0.79	0.05	0.51	0.03	0.69	−0.07	0.33	−0.06	0.41
Q2	0.11	0.12	−0.03	0.73	0.05	0.48	0.06	0.44	−0.11	0.12	−0.02	0.81
Q3	−0.02	0.75	−0.03	0.69	−0.04	0.61	−0.02	0.74	0.02	0.83	−0.14	0.05
Q4	0.06	0.43	0.02	0.74	−0.02	0.74	−0.10	0.16	−0.06	0.39	−0.06	0.44
Q5	0.00	0.95	−0.02	0.75	−0.02	0.74	−0.04	0.59	−0.11	0.13	0.04	0.59
Q6	0.03	0.66	−0.12	0.12	−0.06	0.45	−0.06	0.45	−0.03	0.73	0.06	0.38
Q7	0.03	0.67	−0.06	0.38	0.06	0.38	−0.01	0.90	0.00	0.98	0.06	0.40
Q8	0.20	0.007 **	0.02	0.84	0.13	0.07	0.04	0.62	0.09	0.20	0.10	0.17
Q9	0.18	0.02 *	0.03	0.67	0.08	0.25	0.16	0.03 *	0.16	0.03 *	0.26	0.0003 **
Q10	0.13	0.08	−0.05	0.54	0.05	0.54	0.23	0.001 **	0.23	0.001 **	0.25	0.001 **
Q11	0.19	0.009 **	−0.05	0.46	0.02	0.80	0.05	0.53	0.21	0.005 **	0.22	0.003 **
Q12	0.17	0.02 *	0.08	0.30	0.15	0.04 *	0.25	0.001 **	0.12	0.10	0.08	0.28
Q13	0.05	0.51	−0.06	0.38	0.06	0.38	0.07	0.36	−0.06	0.43	0.06	0.40
Q14	0.14	0.06	−0.06	0.38	0.12	0.10	0.14	0.05	0.00	0.98	0.00	0.95
Q15	0.16	0.03 *	−0.03	0.69	0.11	0.14	0.03	0.67	0.10	0.18	−0.13	0.08
Knowledge domain total	0.11	0.15	−0.05	0.53	0.07	0.38	0.07	0.36	−0.09	0.23	−0.10	0.18
Technique domain total	0.23	0.001 **	0.06	0.41	0.14	0.06	0.21	0.004 **	0.18	0.02 *	0.16	0.03 *
Total	0.23	0.002 **	0.02	0.76	0.12	0.10	0.17	0.02 *	0.09	0.21	0.11	0.14

*p*-values are calculated using Spearman’s rank correlation coefficient. * and ** indicate *p* < 0.05 and *p* < 0.01, respectively. Considering the distribution, categorical variables are converted into binary variables as follows: M = 0, F = 1; N = 0, Y = 1; POAG = 0, non-POAG = 1. ρ, Spearman’s rank correlation coefficient.; HT, hypertension; DM, diabetes mellitus; POAG, primary open angle glaucoma; non-POAG, primary angle closure glaucoma exfoliation glaucoma + other types of glaucoma.

**Table 6:** Multivariate Analysis of the Associations Between SU-GAQ Responses and CGA scores.

Parameter	Knowledge Domain			Technique Domain			Total		
	Estimate	95% CI	*p*-Value	Estimate	95% CI	*p*-Value	Estimate	95% CI	*p*-Value
Intercept	2.32	0.83, 3.81	0.003 **	1.71	−0.26, 3.67	0.09	4.02	1.29, 6.75	0.004 **
Mini-Cog	−0.31	−0.47, −0.16	<0.0001 **	−0.13	−0.33, 0.07	0.21	−0.44	−0.72, −0.16	0.002 **
G8	−0.02	−0.10, 0.06	0.61	−0.07	−0.17, 0.04	0.21	−0.09	−0.23, 0.06	0.23
ACCI	−0.03	−0.14, 0.08	0.59	0.16	0.02, 0.31	0.02 *	0.14	−0.06, 0.33	0.18
Sex (F/M)	−0.04	−0.35, 0.27	0.81	−0.17	−0.58, 0.24	0.42	−0.21	−0.78, 0.36	0.47

*p*-values are calculated using a generalized regression model. In the model, the three CGA test results and sex are included as explanatory variables. * and ** indicates *p* < 0.05 and *p* < 0.01, respectively. CI, confidence interval; ACCI, Age-adjusted Charlson Comorbidity Index; F, female; M, male.

**Table 7:** Eigenvalues and explained variance based on tetrachoric correlations.

Factor	Eigenvalue	Proportion of Variance	Cumulative Proportion
1	7.4	49.2%	49.2%
2	3.3	22.0%	71.2%
3	1.1	7.6%	78.7%

**Table 8:** Varimax-rotated factor loadings for the two-factor model.

Item	Domain	Factor 1	Factor 2
Q1	Knowledge	0.79	0.03
Q2	Knowledge	0.94	0.11
Q3	Knowledge	0.77	0.07
Q4	Knowledge	0.98	0.07
Q5	Knowledge	0.93	0.10
Q6	Technique	0.67	0.57
Q7	Technique	0.36	0.71
Q8	Technique	0.37	0.76
Q9	Technique	−0.06	0.94
Q10	Technique	−0.07	0.85
Q11	Technique	0.06	0.88
Q12	Technique	0.23	0.76
Q13	Technique	0.35	0.71
Q14	Technique	0.46	0.50
Q15	Technique	0.61	0.32

Factor loadings ≥0.40 indicate substantial association with the corresponding factor.

## Data Availability

The raw data supporting the conclusions of this article will be made available by the authors on request.

## Supplementary Materials

The following supporting information can be downloaded at https://www.mdpi.com/article/10.3390/jcm15135203/s1: Table S1: The original Japanese version of the Shimane University Glaucoma Eye Drop Adherence Questionnaire (SU-GAQ); Figure S1: The original printed version of the Shimane University Glaucoma Eye Drop Adherence Questionnaire (SU-GAQ).

## Abbreviations

The following abbreviations are used in this manuscript:

ACCI	Age-Adjusted Charlson Comorbidity Index
CCI	Charlson Comorbidity Index
CGA	Comprehensive Geriatric Assessment
CI	Confidence Interval
DM	Diabetes Mellitus
EFA	Exploratory Factor Analysis
EXG	Exfoliation Glaucoma
FDR	False Discovery Rate
HT	Hypertension
IOP	Intraocular Pressure
IRB	Institutional Review Board
PACD	Primary Angle-Closure Disease
POAG	Primary Open-Angle Glaucoma
SD	Standard Deviation
SU-GAQ	Shimane University Glaucoma Eye Drop Adherence Questionnaire
VF	Visual Field
