# Supplementary material for: Association of Comprehensive Geriatric Assessment with Knowledge- and Technique-Related Eye Drop Adherence Problems in Glaucoma Assessed Using the Shimane University Glaucoma Eye Drop Adherence Questionnaire
Source: J Clin Med. 2026 Jul 3;15(13):5203. doi: 10.3390/jcm15135203 (PMC13362910; doi:10.3390/jcm15135203)
Supplement: Supplementary file 1 [file jcm-15-05203-s001.zip › Figure S1.pdf]

緑内障治療でお困りのことはありませんか?

緑内障治療に関する知識、点眼手技について、お伺いします。
以下の項目であてはまるものに☑チェックしてください。

知識編

- ☐ 緑内障の目薬が、眼圧を下げる目薬であるということを知らない
- ☐ 複数点眼する場合、どれが眼圧を下げる目薬かわからない
- ☐ 複数点眼する場合、点眼間隔を5分以上あけていない
- ☐ 点眼回数を知らない、点眼回数を間違えることがある
- ☐ いつ点眼したらよいかわからない

点眼手技編

- ☐ 点眼時、上を向くことができない
- ☐ 目をしっかり開けることができない
- ☐ 手が震えてうまく点眼できない
- ☐ 点眼位置がずれる
- ☐ 点眼容器の先が見えないため、うまく点眼できない
- ☐ 点眼薬が1回で目に入らないことが多く、何滴も点眼してしまう
- ☐ 点眼薬をさすときに、まつげやまぶたに点眼容器がつくことがある
- ☐ 点眼後、まぶたを閉じる、または、目頭をおさえることができていない
- ☐ 点眼後、目のまわりについた薬液のふき取りや洗顔ができていない
- ☐ 点眼するのを忘れることがある

緑内障の治療を続ける上で、気になることがある方はご記入ください。

監修：谷戸 正樹 先生（島根大学医学部眼科学講座 教授）

Figure S1. The original printed version of the Shimane University Glaucoma Eye-Drop Adherence Questionnaire (SU-GAQ).
