# Supplementary material for: Association of Comprehensive Geriatric Assessment with Knowledge- and Technique-Related Eye Drop Adherence Problems in Glaucoma Assessed Using the Shimane University Glaucoma Eye Drop Adherence Questionnaire
Source: J Clin Med. 2026 Jul 3;15(13):5203. doi: 10.3390/jcm15135203 (PMC13362910; doi:10.3390/jcm15135203)
Supplement: Supplementary file 1 [file jcm-15-05203-s001.zip › Table S1.pdf]

Table S1. The original Japanese version of the Shimane University Glaucoma Eye-Drop Adherence Questionnaire (SU-GAQ)

島根大学緑内障点眼アドヒアランス問診票（SU-GAQ）

**項目No.**	**内容**
**知識関連項目**	
Q1	緑内障の目薬が，眼圧を下げる目薬であると言うことを知らない
Q2	複数点眼する場合，どれが眼圧を下げる目薬かわからない
Q3	複数点眼する場合，点眼間隔を５分以上あけていない
Q4	点眼回数を知らない，点眼回数を間違えることがある
Q5	いつ点眼したらよいかわからない
**手技関連項目**	
Q6	点眼時，上を向くことができない
Q7	目をしっかり開けることができない
Q8	手が震えてうまく点眼できない
Q9	点眼位置がずれる
Q10	点眼容器の先が見えないため，うまく点眼できない
Q11	点眼薬が１回で目に入らないことが多く，何滴も点眼してしまう
Q12	点眼薬をさすときに，まつげやまぶたに点眼容器がつくことがある
Q13	点眼後，まぶたを閉じる，または，目頭を押さえることができていない
Q14	点眼後，目の周りについた薬液の拭き取りや洗顔ができていない
Q15	点眼するのを忘れることがある
